# COVID-19 Parental Vaccine Hesitancy in Romania: Nationwide Cross-Sectional Study

**DOI:** 10.3390/vaccines10040493

**Published:** 2022-03-23

**Authors:** Loredana Sabina Cornelia Manolescu, Corneliu Nicolae Zaharia, Anca Irina Dumitrescu, Irina Prasacu, Mihaela Corina Radu, Adrian Calin Boeru, Liliana Boidache, Irina Nita, Andrei Necsulescu, Cosmin Medar, Corina Marilena Cristache, Razvan Daniel Chivu

**Affiliations:** 1 Department of Microbiology, Parasitology and Virology, Faculty of Midwifery and Nursing, “Carol Davila” University of Medicine and Pharmacy, 050474 Bucharest, Romania; liliana.boidache@yahoo.com (L.B.); irina.nita0910@gmail.com (I.N.); 2 Department of Virology, Institute of Virology “Stefan S. Nicolau”, 030304 Bucharest, Romania; corneliu.zaharia@gmail.com; 3 Department of Fundamental Sciences, Faculty of Pharmacy, “Carol Davila” University of Medicine and Pharmacy, 050474 Bucharest, Romania; ancairinadumitrescu@yahoo.com (A.I.D.); irinaprasacu@yahoo.com (I.P.); 4 Department of Birth, Obstetrics and Gynecology Hospital, 100409 Ploiesti, Romania; calinboeru@yahoo.com; 5 Medical Oncology Department, Elias University Emergency Hospital, 011461 Bucharest, Romania; 6 Department of Emergency, Central Military Emergency University Hospital “Dr. Carol Davila”, 010825 Bucharest, Romania; andrei.neck@gmail.com; 7 Department of Patient Management in the Radiology and Imaging Service, Radiology Techniques, Faculty of Midwifery and Nursing, “Carol Davila” University of Medicine and Pharmacy, 050474 Bucharest, Romania; cosmin.medar@umfcd.ro; 8 Department of Dental Techniques, Faculty of Midwifery and Nursing (FMAM), “Carol Davila” University of Medicine and Pharmacy, 8 Eroilor Sanitari Blvd., 050474 Bucharest, Romania; corina.cristache@umfcd.ro; 9 Department of Public Health and Health Management, Faculty of Midwifery and Nursing, “Carol Davila” University of Medicine and Pharmacy, 050474 Bucharest, Romania; razvan.chivu@umfcd.ro

**Keywords:** COVID-19 vaccination, parental vaccine hesitancy, child vaccination

## Abstract

Background: COVID-19 vaccination started in Romania in December 2020. Child vaccination started in 2021 with children aged 12–15 years in August. For children aged 5–11 years, vaccination started in January 2022. The aim of our study was to describe COVID-19 vaccination hesitancy in Romanian children and vaccine acceptability in the general population. As parental consent is required for child vaccination in Romania, these aspects have a significant association. Methods: An analytical cross-sectional survey was conducted in October and November 2021 during the peak of the 4th COVID-19 wave. Results: After validation, 1645 participants formed the main study group: median age 35 years, 72.83% women, and 35.44% from the medical domain. In total, 1311 (79.70%) participants were vaccinated against COVID-19 and 188 (11.42%) had vaccinated their 12–18-year-old children against COVID-19. Parents’ level of education, geographic area of residence, and COVID-19 vaccination status were significantly associated with COVID-19 vaccination. The hesitancy factors of child vaccination included the novelty of COVID-19 vaccines (62, 47.32%), fear of adverse reactions (32, 24.42%), and anti-vaccinism in general (29, 22.13%). In the studied group, only 188 (11.42%) participants recommended vaccination of 5–11-year-old children. Vaccine acceptability was higher in the general population (1311, 79.70%) than in the medical domain (326 out of 583, 55.91%). General vaccine hesitancy was based mainly on beliefs regarding inefficiency (131, 39.22%) and fears about the side effects of the vaccine (76, 22.75%). Conclusions: Overall, the acceptability of COVD-19 vaccines in the Romanian population was influenced by the level of education, area of residence, and being a COVID-19-vaccinated parent. Public health intervention programs are essential.

## 1. Introduction

For several years, vaccine hesitancy has represented one of the global public health threats [1].

The COVID-19 pandemic has raised vaccine hesitancy to a new level, mostly due to the novelty of the vaccines that have been issued [2].

Parental vaccine hesitancy at international, national, and regional levels may have a great impact on vaccination in general.

COVID-19 vaccination started in Romania in December 2020. A national vaccination campaign was initiated, with the first phase targeting the vaccination of healthcare and social workers [3,4]. As of 28 December 2020, an IT platform, ROVACCINARE, allowed the registration of people for vaccination against COVID-19 (https://programare.vaccinare-covid.gov.ro/; accessed on 20 January 2022). The second phase of the Romanian vaccination campaign targeted people at high risk and the elderly, those aged over 65 years, adults with chronic diseases, and people who are involved in essential activities. The third phase targeted vaccination of the general population and people aged over 16 years, and started on 15 March 2021 [5]. The platform has been operational and allowed the vaccination of children aged 12–15 years old since 1 August 2021 and for individuals receiving the booster (3rd dose) since 28 September 2021 [5]. During the fall of 2021, Romania experienced the fourth wave of COVID-19, with the dominant mutant being the SARS-CoV-2 Delta variant [6]. The peak of new confirmed infections was reached on 18 October with a total of 18,863 new cases. On 2 November, Romania reported 591 deaths on 1 day, with a 7-day average of 454 deaths [5,7].

According to the European Center for Disease Prevention and Control COVID-19 Vaccine Tracker, in Romania, the cumulative uptake of full vaccination in the total population as of 25 January 2022 was 41%, which is still a low value compared with EU/EAA data of 69.7%. The uptake of an additional dose, the 3rd booster dose, in Romania was 7% [7].

On 6 December 2021, the SARS-CoV-2 Omicron variant was detected in Romania, for which the sequencing was performed at the “Cantacuzino” National Institute for Medico-Military Research in Bucharest [8]. Since then, the 5th wave of COVID-19 infection started in Romania and the number of new infections dramatically increased from 349 cases (new confirmed, 25 December 2021) to 34,255 cases (new confirmed, 25 January 2022) [5,9,10], almost 100 times in less than a month.

Although the European Medicines Agency (EMA) recommended that the Pfizer COVID-19 vaccine should be given to children aged 5 to 11 years since 25 November 2021, in Romania, vaccination for this age group only started on 26 January 2022.

Due to the high rate of transmissibility of the Omicron variant, the low rate of vaccination in children of all ages, and for the safety of Romanian children, since December 2021, the Ministry of Health [11] started a testing program in schools using the rapid saliva COVID-19 antigen test. Since then, in Romanian schools, two tests have been required each week, on Mondays and Thursdays.

In Romania, recent studies regarding vaccination, according to the national vaccination program, have shown that a lack of information and fear of side effects has determined parents’ decision to not vaccinate their children in general [12].

Because the vaccination is still the most effective tool against the severe COVID-19 disease, the aim of our study was to assess and discuss COVID-19 vaccination hesitancy in Romanian children because the vaccination of children depends on their parents’ vaccine acceptability. Due to this, we also studies vaccine acceptability in the general population.

## 2. Materials and Methods

### 2.1. Study Design, Sites, and Participants

#### 2.1.1. Study Design

An analytical cross-sectional observational survey was conducted in Romania in the general population. For an accurate design, we used “The Strengthening the Reporting of Observational Studies in Epidemiology” (STROBE) recommendations in our observational study. The STROBE checklist is included in the Supplementary Materials [13].

#### 2.1.2. Study Setting

This study was conducted in Romania in all 41 counties and in the capital, Bucharest, with the aid of volunteers, medical students, and teaching staff from “Carol Davila” University of Medicine and Pharmacy from Bucharest. The students and professors who spread the questionnaire were volunteers and agreed to explain the questionnaire to the public to achieve as many correct answers as possible.

#### 2.1.3. Study Participants

This study was addressed to the general population, all people living in the Romanian counties and the capital. The goal was to collect at least 30 answers from each county. The inclusion criteria for the questionnaire required participants to be 18 years of age or older and living in Romania. The participants were contacted by volunteers, and everybody was welcomed. The domain of activity of the participants was defined as medical and non-medical. The medical domain of activity included medical doctors, nurses, pharmacists, paramedics, midwives, and medical students. We included this category because the medical domain responders may have influence over the general population regarding vaccination, and they are a key factor in vaccine importance education. The non-medical domain included all types of professions unrelated to the medical ones. The level of education was reflected by the level of completed studies, including high school, post-secondary school, and university/post-university studies.

### 2.2. Survey Questionnaire and Data Collection

Data were collected with the use of a self-administered questionnaire. The questionnaire was administered online for 2 months: October and November 2021, during the peak of the COVID-19 4th wave (and with the known threat of the emergence of the 5th COVID-19 wave), and because of the epidemiological situation, people were eager to help in any way to resolve the COVID-19 situation, resulting in a good response rate. The questionnaire (which can be found in the Supplementary Materials File S1) comprised 19 multiple choice questions divided into sections (general information and information about the COVID-19 vaccine). It was distributed by volunteers through media messages, both individually and in institutional communication groups in the form of a link. After it was issued in Google Forms, informed consent was required and obtained from each willing participant, and the responder was initially informed of the purpose of the survey. The questionnaire could only be submitted after all its questions were answered.

### 2.3. Ethical Approval

The questionnaire was peer-validated and approved by the Ethics Committee of the Obstetrics and Gynecology Hospital, Ploiesti, Romania (14325/21.12.2020); all the procedures in the study respected the ethical standards of the Helsinki Declaration. Informed consent was compulsory.

### 2.4. Statistical Analysis

For statistical analysis, we used the Microsoft Office package Excel and IBM^®^ SPSS^®^ Statistics Version 23.0 software. For data processing, the COUNTIFS function in Excel was used to filter and sort the initial database. Categorical variables on the sociodemographic characteristics of the parents were expressed using descriptive statistics and frequencies. The chi-squared test (for categorical variables) and measures of association (ϕ-test) were used to determine the parental characteristics associated with their willingness to vaccinate children under the age of 18 years with the COVID-19 vaccine.

We used logistic regression to analyze the factors (level of education, geographic area, vaccination status of the parent) that influenced the odds of children being vaccinated. The level of significance was set to 0.05.

## 3. Results

In total, 1650 individual answers were gathered. The answers to the questions are summarized in the Supplementary Materials File S2. The first part of the survey targeted general data to characterize the group. We excluded five participants due to incomplete answers regarding their age, vaccine status, and profession. After validating the rest, 1645 participants remained. The mean age of the participants was 34.91 ± 12.58 years (limits: 18–77), with a median of 35 years, and 1198 (72.83%) of the participants were women. Of our group of 1645 participants, 583 (35.44%) were from the medical domain. Most of the participants in the survey lived in an urban geographic area (1343, 81.64%) and had completed post-university studies (740, 59.63%). The general characteristics of the survey participants are summarized in Table 1.

In the whole study group, 1311 (79.70%) participants were vaccinated against COVID-19 but only 188 (11.42%) participants had vaccinated their children aged from 12–18 years, as shown in Table 2. Our survey was conducted during a period when COVID-19 vaccination of children aged 5–11 years was not available in Romania.

Some of the study variables, after stepwise selection, were significantly associated with COVID-19 vaccination of children aged between 12 and 18 years. From the logistic regression analysis, level of education (ORa = 0.546; 95% CI: (0.321; 0.927)), geographic area of residence (ORa = 0.669; 95% CI: (0.369; 1.215)), and being a COVID-19-vaccinated parent (ORa = 0.032; 95% CI: (0.013; 0.082)) (Table 3) were significantly associated with willingness to vaccinate children.

Between 26.7% and 35.7% of variance of the child vaccination is explained by the following variables: level of education of the parent, geographic area, and vaccinated/non-vaccinated parent (Table 4).

Because children aged between 12 and 18 years need their parents’ consent for immunization in Romania, we considered the discovery of all factors that may influence the decision to vaccinate a child against COVID-19 critical. In our study group, 792 (48.15%) participants had children, but out of them, only 319 (19.39%) participants had children aged between 12 and 18 years. The remaining 473 (28.75%) participants had younger children. In total, 188 (11.42%) participants matched all the discussed criteria, and their children were aged between 12 and 18 years and were immunized against COVID-19. Vaccination was also recommended for children aged 5 to 11 years when the vaccination was available, as shown in Table 5. The variables that were significantly associated with COVID-19 vaccination in the 12–18-years age group were also used in the cases of children aged between 5 and 11 years: level of education (ORa = 0.494; 95% CI: (0.313; 0.779)), geographic area of residence (ORa = 0.591; 95% CI: (0.355; 0.984)), and COVID-19 vaccination status of the parent (ORa = 31.440; 95% CI: (12.336; 80.128).

Among the participants who had children in the 12–18-years age group and did not vaccinate their children against COVID-19, 131 (7.96%) participants stated the following reasons for not immunizing their children: they considered the COVID-19 vaccines too new and that they needed to be studied more (62, 47.32%), they were afraid of the adverse reactions of the COVID-19 vaccines (32, 24.42%), they did not agree with vaccination in general (29, 22.13%), or they believed that is better to be immunized naturally, i.e., by being infected and having COVID-19 disease (8, 6.10%).

Regarding the vaccine acceptability in the general population, as shown in Table 1, we believe that the Romanian population understood the important need for COVID-19 vaccination. Out of 1645 participants, 1311 (79.70%) were immunized. In our study, vaccine acceptability was higher in the general population (79.70%) than among healthcare workers from the medical domain (55.91%), with 326 out of 583 indicating acceptability. The Romanian population have access to all types of approved COVID-19 vaccines in the European Union. Most of the vaccinated participants (1104, 84.21%) were immunized with mRNA vaccines. Out of the 1311 vaccinated participants, 482 (36.76%) experienced adverse reactions after receiving the vaccination, such as pain at the site of injection, inflammation at the site of injection, fever, and/or chills. The factors underlying vaccine hesitancy among adults included the belief that the vaccine was not effective (131, 39.22%), fears regarding the side effects of the vaccine (76, 22.75%), the belief that it is more useful to make antibodies by acquiring the disease than being vaccinated (57, 17.06%), disagreeing with vaccination in general (35, 10.47%), and passing through the COVID-19 disease (11, 3.29%). The characteristics of the two groups: COVID-19-vaccinated and non-COVID-19-vaccinated participants, are presented in Table 6. There were no statistical differences between the two groups.

After 6 months of complete immunization, the Romanian population could receive a third booster dose. In our group, from the 1311 COVID-19-vaccinated participants, 404 (30.81%) received the 3rd booster dose. All participants with the booster dose received an mRNA COVID-19 vaccine. The remaining participants declared either that they had no intention of receiving the booster dose (405, 30.89%) or that they will do it in the future (502, 38.29%). Most of the 404 participants that received the 3rd booster dose developed a minor adverse reaction, such as pain at the site of injection (341, 84.40%) or enlargement of the axillary lymph nodes (63, 15.59%).

## 4. Discussion

The COVID-19 vaccine could represent the best hope to end the pandemic and a key factor in resolving the sanitary crisis. Scientists have competed in the development of vaccines in unprecedented joint efforts within the scientific community. However, as seen in the context of other diseases in humans, agriculture, and wildlife, the challenge of eradicating the spread of a disease does not end with the discovery of an effective vaccine. The implementation of the vaccination program is extremely important. Here, we aimed to assess the acceptability of the COVID-19 vaccine in the Romanian adult population and the willingness of Romanians to vaccinate their children as the COVID-19 vaccines became available in Romania for different age groups, thus documenting COVID-19 vaccination in Romanian children. We also explored the socio-demographic variables of the vaccinated and non-vaccinated participants in our studied groups and their COVID-19 vaccine beliefs.

At the global level, studies have highlighted a hesitancy vaccinate children using different vaccine types in general [14,15,16,17,18,19,20]. This hesitancy to vaccinate translates to severe consequences. Moreover, beliefs in conspiracy theories, distrust in science and decision-makers, and distrust in official channels of information may also increase hesitation regarding vaccination. Understanding their causes could improve our ability to respond to the pandemic. Various impressions of adverse events associated with vaccines have been experienced in Romania, which are mostly scandals based upon undocumented fears, leading to a loss of public confidence in vaccines [21,22].

Several studies have warned against misinformation against COVID-19 vaccines, which can be easily spread through social media, requiring collaborative efforts by governments, health decision-makers, and media sources to spread clear and timely messages that support the safety and efficacy of currently available COVID-19 vaccines. This promotes vaccines and builds confidence in COVID-19 vaccination among the general public [23,24,25,26].

We believe the same happened here as 22.75% (76 out of 334), of the unvaccinated participants reported concerns about the possible side effects of the COVID-19 vaccine. Some parents questioned the need to vaccinate their children, which was often based on medical events temporarily associated with the vaccination, which happened to family members or friends/acquaintances or were reported in newspapers, TV shows, the internet, etc. Anti-vaccination groups are widely publicized and attempt to convince parents that avoiding vaccination is safe and effective [21,22,27,28].

Social networks must not harm the prevention and control of infectious diseases. Their potential can be exploited with public health interventions, reaping significant benefits. In the case of vaccination, in Romania, social media platforms have become an important vector in providing accurate scientific information. For example, Romania has the largest Facebook group in the world, which provides parents vaccination tips directly from primary care physicians [29,30]. Given this, the use of such social media platforms will prove beneficial in addressing the vaccine hesitation in Romania, thus helping to implement any potential anti-COVID-19 immunization strategy.

In Romania, by the end of December 2020, there were over half a million documented SARS-CoV-2 infections. The actual figures probably far exceeded this figure due to insufficient testing [31]. Despite these data, our study showed a good level of acceptability of the COVID-19 vaccination among the population under study (1311, 79.69%) but a low level of vaccinated children (188, 11.42%). This justifies the low figures indicating about 8 million vaccinated people, with only 2 million people obtaining the booster dose and only 2113 vaccinated children, according to the ROVACCINARE-Platform [5]. Previous research has shown that high population sensitivity and high-risk perception of the disease are associated with better pandemic control [32]. Thus, prevention and control measures should be encouraged. The perceived barriers against COVID-19 immunization found in this study, namely concerns about side effects and the efficacy of the vaccine, have also been reported in other studies related to the introduction of a new vaccine [33]. This study was conducted just before the emergence of the Omicron strain, a strain that, according to studies, affects a younger population. Studies have shown that children generally develop mild symptoms and can experience robust effects with high levels of SARS-CoV-2 replication, thus significantly contributing to viral spread [34].

In this study, despite a large proportion of participants (872, 53.01%) expressing an intention to vaccinate their children, a significant percentage did not have this intention (773, 46.99%). Uncertainties about the safety of the vaccine, and the efficacy and benefits of vaccinating children were the most widespread reasons cited for parents’ refusal of the COVID-19 vaccine.

Several recent studies showed similar levels of vaccine acceptability in the general population, such as 71.4% (1878 responders) in a study from Mozambique [35] vs. 79.70% (1645 responders) in our study. Some differences regarding the reasons underlying vaccine hesitancy include fears about the vaccine’s side effects (29.6% in the Mozambique study vs. 22.75% in our study) and the belief that the vaccine is not effective (52% in the Mozambique study vs. 39.22% in our study). In the Mozambique study, vaccine acceptability was relatively high among healthcare workers but significantly lower in the rest of the population. This finding was different from our study, where vaccine acceptability in the medical domain was 55.91% lower than in the general population (79.70%), as reported in Romanian studies [4,35]. The Romanian population is very cautious when it comes to science. In our study, most of the participants did not trust COVID-19 vaccines, as they considered COVID-19 vaccines have not been adequately researched (62, 47.32%), which was not encountered in either Polish or Saudi Arabian studies [36,37]. Another predictor of vaccine hesitancy appears to reside in beliefs in conspiracy theories [36,38], which was not encountered in our study. Findings from UK studies revealed that age and ethnicity were the only sociodemographic factors to predict vaccine hesitancy, which was again different from our findings [39].

Similar to Italian studies in the Romanian population, mRNA vaccines were preferred for the initial vaccination and booster dose [40].

The COVID-19 pandemic has disrupted the lives of millions of children across the world for several reasons. The COVID-19 vaccination could end this [41,42] with the help of parents because in Romania, child vaccination is only possible with parents’ permission, so vaccination’s acceptability in the general population is critical.

Studies from Australia, conducted at the same time as our study [43], showed that vaccine intention increased in the last months of 2021 in the general population but not among children. Similar to our findings, parents who intended to vaccinate themselves (OR: 0.599, 95% CI: (0.367–0.980)) reported greater acceptance of their children being vaccinated in a Saudi Arabia study [44].

From our findings, the level of education of participants (ORa = 0.494; 95% CI: (0.313; 0.779)) was significantly associated with COVID-19 vaccination in children aged between 5 and 11 years, which is similar to other findings [45] and contrary to others [46].

In children of all ages, either the 12–18-year or 5–11-year groups, vaccine hesitancy has been identified all over the world [47,48].

### Limitation of the Study

This study was subject to several potential limitations. Firstly, this study was limited by its cross-sectional design. Consequently, the representativeness of the study sample is limited due to the default selection of respondents who have digital skills as the studied sample only contained people who could use the internet and electronic documents. This is also supported by the age of the respondents, who had a median age of 35 years, and the questionnaire format, which was a Google Form questionnaire that was accessed by a link and electronically self-administered. Secondly, another limitation of this study is that it only analyzed only a short time period of 2 months, Octobe and November 2021, and included a limited number of participants with children (792, 48.15%). It is possible that people’s acceptability of child vaccination may change over time as major news about vaccines appears daily, and political circumstances and epidemiological data change; therefore, longitudinal monitoring is indicated. Third, there are some limitations regarding the place of residence, as 81.64% of our studied group lived in an urban area, and 35.44% of the studied participants worked in the medical domain. Nevertheless, this may not, in fact, be a limitation because these people are opinion formers and may support children’s vaccination.

## 5. Conclusions

The findings of this study suggest a high perception of barriers preventing and a low perception of benefits in receiving the COVID-19 vaccines in Romanian children. The factors that influenced children’s vaccination included the level of education, the area of residence, and the vaccination status of the parent. Child vaccination hesitancy was driven by the novelty of COVID-19 vaccines, fears about adverse reactions, and anti-vaccinism in general. Overall, the acceptability of COVD-19 vaccines in the Romania population was influenced by the level of education and area of residence. The vaccine’s acceptability in the medical domain was lower than in the general population. General vaccine hesitancy was based mainly on the beliefs about inefficiency and fears about the side effects of the vaccine.

Therefore, public health intervention programs that focus on raising the awareness of the benefits of vaccination and reducing identified barriers are essential. It is therefore important to provide adequate information to the public, particularly to explain the importance of COVID-19 vaccination and to provide sound evidence regarding the safety and efficacy of the vaccine to support personal and media coverage.

## Figures and Tables

**Table 1:** Vaccination/infectious COVID-19 status, socio-demographic, and educational characteristics of the respondents to the questionnaire.

Characteristics	*N* = 1645
Vaccinated against COVID-19	1311 (79.70%)
Not infected with SARS-CoV-2	581 (35.32%)
Gender (women)	1198 (72.83%)
Age (median, min-max)	35 (18–77)
Geographic area (urban)	1343 (81.64%)
Family status: married	763 (46.38%)
Domain of activity: medical	583 (35.44%)
Level of education	
High school	487 (29.60%)
Post-secondary school	109 (6.63%)
University/post-university studies	1032 (62.74%)
Have children	792 (48.15%)

**Table 2:** Vaccination/infectious COVID-19 status, socio-demographic, and educational characteristics of respondents who had vaccinated their children (12–18 years).

Characteristics	*N* = 188
Not infected with SARS-CoV-2	124 (65.96%)
Gender (women)	144 (76.6%)
Age (median, min-max)	46 (28–68)
Geographic area (urban)	157 (83.51%)
Family status: married	150 (79.79%)
Vaccinated against COVID-19	183 (97.34%)
Level of education	
High school	17 (9.04%)
Post-secondary school	18 (9.57%)
University/post-university studies	150 (79.79%)
Domain of activity: medical	80 (42.55)

**Table 3:** Logistic regression model for COVID-19 vaccination of children aged between 12 and 18 years.

	B	E.S.	Wald	*p*	ORa	Lower CI 95%	Upper CI 95%
COVID-19 vaccination status of the parent	−3.440	0.480	51.447	0.000	0.032	0.013	0.082
Level of education (university and more vs. high school and secondary school)	−0.606	0.271	5.014	0.025	0.546	0.321	0.927
Geographic area (urban vs. rural)	−0.401	0.304	1.740	0.187	0.669	0.369	1.215
Constant	0.934	0.155	36.272	0.000	2.544		

**Table 4:** Variance of child vaccination.

Cox and Snell R Square	Nagelkerke R Square
0.267	0.357

**Table 5:** Logistic regression model for COVID-19 vaccination of children aged between 5 and 11 years.

	B	E.S.	Wald	*p*	ORa	Lower CI 95%	Upper CI 95%
Level of education (university and more vs. high school and secondary school)	−0.706	0.233	9.208	0.002	0.494	0.313	0.779
Constant	0.324	0.122	7.004	0.008	1.383		
	B	E.S.	Wald	*p*	ORa	Lower CI 95%	Upper CI 95%
Geographic area (urban vs. rural)	−0.526	0.260	4.081	0.043	0.591	0.355	0.984
Constant	0.231	0.115	3.999	0.046	1.259		
	B	E.S.	Wald	*p*	ORa	Lower CI 95%	Upper CI 95%
COVID-19 vaccination status of the parent	3.448	0.477	52.184	0.000	31.440	12.336	80.128
Constant	−2.760	0.461	35.821	0.000	0.063		

**Table 6:** Characteristics of COVID-19-vaccinated and non-vaccinated participants in the survey.

Characteristics	COVID-19 Vaccinated *N* = 1311; 79.69%	COVID-19 Not Vaccinated *N* = 334; 20.30%
Age		
Mean	35.44	32.84
Standard Deviation	12.82	11.38
Median (Min/Max)	35 (18/77)	35 (36/63)
Gender		
Women	931 (71.01%)	236 (70.65%)
Man	350 (26.69%)	98 (29.34%)
Geographic area		
Urban	1086 (82.83%)	259 (77.54%)
Rural	225 (17.16%)	75 (22.45%)
Family status		
Married	616 (46.98%)	147 (44.01%)
Single	695 (53.01%)	187 (55.98%)
Level of education		
Lower	473 (36.07%)	141 (42.21%)
high	838 (63.92%)	193 (57.78%)
Infected with SARS-CoV2		
Yes	413 (31.50%)	166 (49.70%)
No	898 (68.49%)	168 (50.29%)
Domain of activity		
Medical	326 (24.86%)	257 (76.94%)
Other	985 (75.13%)	77 (23.05%)
Do you have children?		
Yes	642 (48.97%)	150 (44.91%)
No	669 (51.02%)	184 (55.08%)

## Supplementary Materials

The following supporting information can be downloaded at: https://www.mdpi.com/article/10.3390/vaccines10040493/s1, File S1: vaccination questionnaire; File S2: questionnaire response; File S3: STROBE-checklist.
