# Supplementary material for: COVID-19 Parental Vaccine Hesitancy in Romania: Nationwide Cross-Sectional Study
Source: Vaccines (Basel). 2022 Mar 23;10(4):493. doi: 10.3390/vaccines10040493 (PMC9024952; doi:10.3390/vaccines10040493)
Supplement: Supplementary file 1 [file vaccines-10-00493-s001.zip › Supplimentary_material_1_Questionnaire.pdf]

DO I GET VACCINATED ANTI COVID-19? COVID-19 VACCINATION STUDY AND THE FEARS RELATED TO THIS VACCINATION

Hello!

We invite you to contribute to our research on COVID-19 vaccination and the fears associated with this vaccination among the Romanian population. The questionnaire to be completed has several sections and is addressed to the general population. The completion time is about 10 minutes. Participation is voluntary and your answers will be treated confidentially, the information you provide will only be used for research purposes. You have the freedom at any time to withdraw from the study, to interrupt or to resume the questionnaire.

The research respects the international ethical recommendations regarding the absolute confidentiality of the data collected in the study as well as the anonymity and security of the data of the respondents. Also, this research respects the requirements of EU Regulation 2016/79, on the protection of individuals with regard to the processing of personal data and the free movement of such data and of Law no. 506/2004, on the processing of personal data and the protection of privacy in the electronic communications sector.

It is important that during the study you answer the questions as honestly as possible in order to draw the right conclusions.

For any clarification related to this study, during or after its completion you can contact us by e-mail at **loredana.manolescu@umfcd.ro**

I. GENERAL INFORMATION

1. What profession do you have? *
Your answer:……….

2. Gender: *
male
female

3. Age in years: *
Your answer:………

4.Last completed studies: *
general school
high school
post-secondary school
university and / or postgraduate studies

5. Residence: *
rural
urban

6. Family status: *
married
unmarried

7. Do you have children? *

yes
not

8. How old are your children?
Your answer:……….

II. COVID-19 VACCINATION INFORMATION

1. Have you been vaccinated against Covid-19? *
yes
not

2. If not, why did you choose not to vaccinate? *

I do not agree with vaccination
I'm afraid of side effects
I think it's better to get antibodies by disease than by vaccination
I think this vaccine is far too new, more studies are needed
I'm vaccinated
Other:…………

3. If you have been vaccinated, how long has it been since you were completely vaccinated against COVID 19? (The full vaccination schedule is considered to be two doses for Pfizer-BioNTech, Moderna, Astra Zeneca vaccines and only one dose for Johnson & Johnson vaccine): *
a month or less
less than 6 months
more than 6 months
I didn't get vaccinated

4. What vaccine / vaccination schedule did you do? *
Pfizer-BioNTech
Moderna
Oxford-Astra-Zeneca
Johnson & Johnson
I didn't get vaccinated
heterologous vaccination (you have combined the vaccines)

5. Did you do the dose booster according to the initial schedule? (dose booster is considered to be the third dose with Pfizer-BioNTech or Moderna for all vaccines except Johnson & Johnson then it is the second dose) *
Yes
I didn't do it but I will
I didn't do it and I won't do it

6. With what vaccine did you do the dose booster? *
Pfizer-BioNTech
Moderna
I didn't do the dose booster

7. Did you experience side effects as a result of the dose booster? *
Yes, local pain in the area where the vaccine was given
Yes, fever
Yes, chills/
Yes, temporary facial paralysis
Yes, inflammation of the lymph nodules/nodes under the arm (axillary lymph nodules/nodes)
Yes, other symptoms
No, I didn't have any side effects
No, I'm not vaccinated at all
I haven't done the dose booster yet

8. If you have children between the ages of 12 and 18, have you vaccinated your children against COVID-19? *
yes
not
I have no children between the ages of 12 and 18

9. If you have not vaccinated your children over the age of 12, what is the reason? *
I do not agree with vaccination
I'm afraid of side effects
I think it's more useful to make antibodies by disease than by vaccination
I think this vaccine is far too new, more studies are needed
Other (specify) ....
I vaccinated the child / the children
I do not have children over 12 years old

10. Have you been infected with SARS CoV2? Did you get COVID-19? *
Yes, before the first dose of vaccine
Yes, between dose I and dose II of the vaccine
Yes, between dose II and dose III of the vaccine
No, so far
Yes, but I'm not vaccinated

11. For whom do you recommend anti-COVID-19 vaccination? *
Pregnant women
Elderly people
Staff from socio-medical institutions and public interest
Children over 12 years old
To all persons for whom the vaccine leaflet indicates vaccination
I do not recommend anti - COVID-19 vaccination
Children between the ages of 5 and 12 when vaccination for this category of population will be approved
Other:……
