# Supplementary material for: COVID-19 Parental Vaccine Hesitancy in Romania: Nationwide Cross-Sectional Study
Source: Vaccines (Basel). 2022 Mar 23;10(4):493. doi: 10.3390/vaccines10040493 (PMC9024952; doi:10.3390/vaccines10040493)
Supplement: Supplementary file 1 [file vaccines-10-00493-s001.zip › Supplimentary_material_2_Questionnaire_response.pdf]

Characteristic	N = 1645	
**Sex**	Feminine	1198 (72.83%)
	Masculine	447 (27.17%)
**Age**	34 (IQR: 23, 45)	
**Geographic area**	Rural	302 (18.36%)
	Urban	1343 (81.64%)
**Studies**	Post university /University studies	1032 (62.74%)
	College	487 (29.60%)
	Post-secondary school studies	109 (6.63%)
	General studies	17 (1.03%)
**Family status**	Married	763 (46.38%)
	Unmarried	882 (53,62%)
**Field of activity:**	Medical	113 (6.87%)
	Other	1532 (93.13%)
**People with children**	792 (48.15%)	

Question	yes	no
Have you been infected with Covid-19?	581 (35.32%)	1064 (64.68%)
Have you been immunized with COVID-19 vaccine?	1311 (79.70%)	334 (20.30%)
Have you had any side effects from the vaccination?	482 (36.76%)	550 (63.24%)
Do you have family members who are or have been infected with COVID-19?	534 (52,3%)	487 (47,70%)
Did you do the dose booster according to the initial schedule?	1240 (75.38%)	405 (24.62%)

If you have children between the ages of 12 and 18, have you vaccinated your children against COVID-19?	202 (12.28%)	179 (10.88%)

If you have not vaccinated your children over the age of 12, what is the reason?

I do not agree with vaccination	29 (6.9%)
I'm afraid of side effects	32 (7.62%)
I think it's more useful to make antibodies by disease than by vaccination	21 (5%)
I think this vaccine is far too new, more studies are needed	62 (14.76%)
Other (specify)	56 (13.33%)
I vaccinated the child / the children	220 (52.38%)

What vaccine / vaccination schedule did you do?	Pfizer-BioNTech	1008 (61.28%)
	Johnson & Johnson	101 (6.14%)
	Oxford-Astra-Zeneca	103 (6.26%)
	Moderna	95 (5.78%)
If you have not been vaccinated, the reason is:	I do not agree with vaccination	35 (10.48%)
	I think this vaccine is far too new, more studies are needed	131 (39.22%)
	I'm afraid of side effects	76 (22.75%)
